# Regeneration of collecting lymphatic vessels following injury

**DOI:** 10.21203/rs.3.rs-3025656/v1

**Published:** 2023-07-03

**Authors:** Mohammad S. Razavi, Pin-Ji Lei, Zohreh Amoozgar, Nichaluk Leartprapun, Seemantini K. Nadkarni, James W. Baish, Timothy P. Padera, Lance L. Munn

**Affiliations:** 1 Edwin L. Steele Laboratories, Department of Radiation Oncology, Massachusetts General Hospital Cancer Center, Massachusetts General Hospital and Harvard Medical School, Boston, MA 02114, USA; 2 Wellman Center for Photomedicine, Massachusetts General Hospital, Harvard Medical School, Boston, MA 02114 USA; 3 Biomedical Engineering, Bucknell University, Lewisburg, PA 17837, USA

**Keywords:** Lymphatic regeneration, Collecting lymphatic vessels, Lymphatic injury, Lymphatic pumping, Lymphedema

## Abstract

Secondary lymphedema is a debilitating condition driven by impaired regeneration of lymphatic vasculature following lymphatic injury, surgical removal of lymph nodes in cancer patients or infection. However, the extent to which collecting lymphatic vessels regenerate following injury remains unclear. Here, we employed a novel mouse model of lymphatic injury in combination with state-of-the-art lymphatic imaging to demonstrate that the implantation of an optimized fibrin gel following lymphatic vessel injury leads to the growth and reconnection of the injured lymphatic vessel network, resulting in the restoration of lymph flow to the draining node. Intriguingly, we found that fibrin implantation elevates the tissue levels of CCL5, a potent macrophage-recruiting chemokine. Notably, CCL5-KO mice displayed a reduced ability to reconnect injured vessels following fibrin gel implantation. These novel findings shed light on the mechanisms underlying lymphatic regeneration and suggest that enhancing CCL5 signaling may be a promising therapeutic strategy for enhancing lymphatic regeneration.

## One Sentence Summary

Implantation of fibrin gel promotes lymphatic regeneration after injury by elevating the chemokine CCL5 in tissue.

## Introduction

The lymphatic system is key for tissue fluid homeostasis, immune surveillance, and lipid and macromolecule transport (1). The lymphatic system relies on the contractile activity of collecting lymphatic vessels to effectively transport lymph (2). Collecting lymphatic vessels have specialized lymphatic muscle cells (LMCs) that drive lymphatic contractions and intraluminal valves to ensure unidirectional lymph propulsion (2). Most lymphatic endothelial cells (LECs) arise embryonically from the cardinal vein, a process that is guided by lymphangiogenic factors such as VEGFC (3, 4). Such lymphangiogenic pathways are known to substantially decrease in adults, but can be reactivated in pathological scenarios, such as cancer and inflammation (5, 6).

In clinical settings, the lymphatic vasculature is routinely damaged due to trauma, cancer surgery and therapy, and secondary to infection (7–9). Such lymphatic damage and subsequent pumping dysfunction cause lymphedema, characterized by tissue swelling, chronic inflammation, fibrosis, and recurrent infection (10). Lymphedema is prevalent among cancer survivors, but unfortunately, there is no cure or even FDA-approved drug for lymphedema, and therapeutic options are limited. (10, 11). Some surgical interventions, such as lymphaticovenous anastomosis and vascularized lymph node transfer, have shown some promise to reduce the volume of edema (12–14). The underlying mechanisms that underpin these successes are being actively investigated (15, 16).

Recent research suggests that immune cells play a crucial role in lymphangiogenic responses and collateralization following injury (17, 18). Macrophages secrete factors known to be important in cancer and inflammation-associated lymphangiogenesis (19–22). More recently, CD4+ T lymphocytes have been proposed to inhibit the regeneration of lymphatic vessels by secreting anti-lymphangiogenic cytokines such as IFN-γ, TGFβ, IL-4, and IL-13 (23). The inhibition of lymphocyte infiltration following lymphatic damage has also led to improved lymphatic collateralization and lymphatic contraction in mice (23). Leukotriene B4 (LTB4), a mediator of inflammation that is elevated in lymphedema patients, has also been found to promote lymphatic sprouting at low doses while causing LEC damage and apoptosis at higher concentrations (24). In addition, cytokines such as TNF-α and IL-1α have been shown to recruit cells secreting lymphangiogenic factors (25–27). Strategies to promote lymphatic regeneration, such as gene and growth factor therapies, the application of natural and synthetic hydrogels, and engineering lymphatic vasculature, have also gained attention (28, 29). In mice, a combination of lymph node transplantation and VEGF-C therapy has been shown to repair the damaged lymphatic vasculature after lymph node resection (30). The application of fibrillar collagen following node resection has also improved lymphatic density at the injury site (31). Seeding LECs with other cell types, such as fibroblasts or adipocyte-derived cells, in fibrin and collagen hydrogels can also promote the growth of new lymphatic capillaries (32, 33).

Despite these efforts, the regeneration and repair of collecting lymphatic vessels remain poorly understood, and the mechanisms underlying such regeneration and repair responses remain largely unexplored. Toward this end, we sought to answer whether fibrin scaffolding promotes the regeneration and repair of collecting lymphatics following collecting vessel injury. We found that collecting lymphatic vessel explants cultured in 3D fibrin hydrogel, in the absence of growth factors such as VEGF-C, promotes lymphatic sprouting *in vitro*. We developed a novel surgical mouse model to study lymphatic regeneration and showed that collecting vessels that have had a segment resected can regenerate sufficiently following fibrin implantation to restore lymphatic flow to the draining lymph node. We also found that macrophages infiltrate the tissue surrounding regenerating lymphatic vessels and that macrophage depletion via clodronate liposomes abrogates lymphatic regeneration. Further, the lymphatic regeneration after fibrin implantation was accompanied by an increase in CCL5, a chemokine that recruits macrophages. Finally, genetic mouse models showed that the CCL5-CCR5 axis, as well as other signaling pathways that recruit macrophages (the CX3CR1-CX3CL1 axis and the CCR2-CCL2 axis), play an important role in collecting lymphatic vessel regeneration.

## Results

### Fibrin gel promotes the sprouting of collecting lymphatic vessels *in vitro* in a fibrinogen concentration-dependent manner.

**Figure 1.**
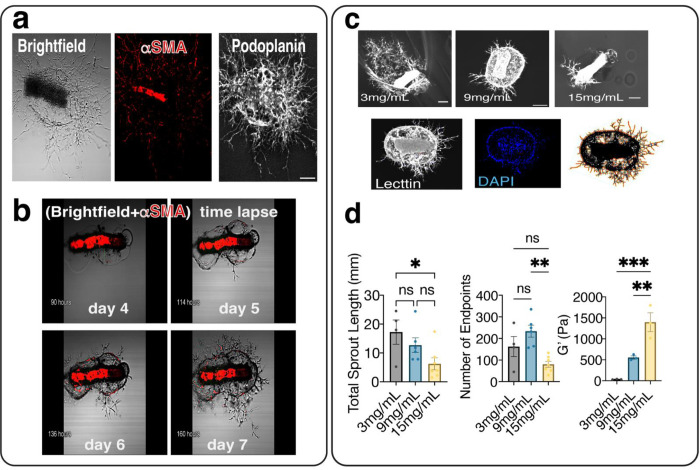
Fibrin gel supports lymphatic sprouting in vitro, and the sprouting response varies with fibrinogen concentration. **a)** Representative image of a segment of a collecting lymphatic vessel embedded in fibrin gel; phase-contrast imaging shows the vessel explant and sprouts in fibrin gel; PDPN (Alexa-647, white) indicates lymphatic endothelial cells; αSMA (Cy3, red) indicates lymphatic muscle cells. **b)** Representative time-lapse imaging shows the time course of sprout formation, LMC migration (DsRed- αSMA+ cells), and fibrin gel degradation. **c)** Representative images of sprouts stained with lectin (DyLightTM 649) at different fibrinogen concentrations show that sprouting changes as fibrinogen concentration increases (3 mg/mL, 9 mg/mL, and 15 mg/mL fibrinogen concentrations). A representative 3D reconstruction of sprouts at a concentration of 9 mg/mL with DAPI staining including a z-projection image, image segmentation and sprout quantification. **d)** Sprouting metrics (total length of sprouts and total number of endpoints) were quantitatively analyzed with the AngioTools software. Quantification of the viscoelastic properties of fibrin gel at different concentrations reveals that the elastic storage modulus (G’) increases significantly with increasing fibrin concentration. One-way ANOVA analysis with a Tukey post hoc test was used to determine whether there was a statistically significant difference between different groups (*p < 0.05, **p < 0.01, ***p < 0.001). The scale bar indicates 200μm. All data were presented as mean (±s.e.m.).

Segments of collecting lymphatic vessels (~0.5 mm) were excised from the flanks of mice and cultured in a 3D fibrin scaffold using Opti-MEM media supplemented with 4% Ultroser G (a chemically semi-defined serum without VEGF-C) and under 5% O_2_ and 5% CO_2_ conditions. Only segments having outgrowths were used for quantitative analysis. When cultured in fibrin gel, lymphatic vessels sprouted over time (Figure 1a). Immunostaining demonstrates that lymphatic sprouts express podoplanin (PDPN) and α-SMA, indicating that both endothelial cells and lymphatic muscle cells contribute to the sprouts. Time-lapse imaging also shows that on day 5, LMCs start migrating along sprouts and that the fibrin gel breaks down as sprouts extend around the explant. (Figure 1b, and **Supplementary Video 1**). The sprouting response changes as fibrinogen concentration increases (Figure 1c). Quantitative analysis of sprouts revealed that total sprout length significantly decreases with increasing fibrinogen concentration (n = 3 distinct vessels in each condition), from 17.23±4.21mm for 3mg/mL to 6.27±2.07mm for 15mg/mL (Figure 1d). There is a statistically significant difference in the total number of sprout endpoints between 9 mg/mL (233.7±27.2) and 15 mg/mL (77.5±16), while the average endpoint number for 3 mg/mL (162.5±46.5 mm) has the highest mean standard error and does not significantly differ from that of 9 mg/mL and 15 mg/mL (Figure 1d). Rheological testing also revealed that the gel’s viscoelastic properties changed significantly with an increase in fibrinogen concentration (Figure 1d). The storage modulus G′ was 555.10±38.71 (Pa) for 9mg/mL compared to 34.76±3.86 (Pa) for 3mg/mL and 1397.33±225.73 (Pa) for 15mg/mL, respectively (n=3). Vessels were only cultured for six days prior to analysis, as the results of vessel sprouting become highly variable after this point due to fibrin gel degradation. Based on explant sprouting responses, gel stability and mechanical properties at different fibrinogen concentrations, a 9 mg/mL concentration was chosen for *in vivo* implantation.

### Fibrin gel implantation promotes collecting vessel regeneration and restores lymphatic transport to the draining lymph node.

**Figure 2.**
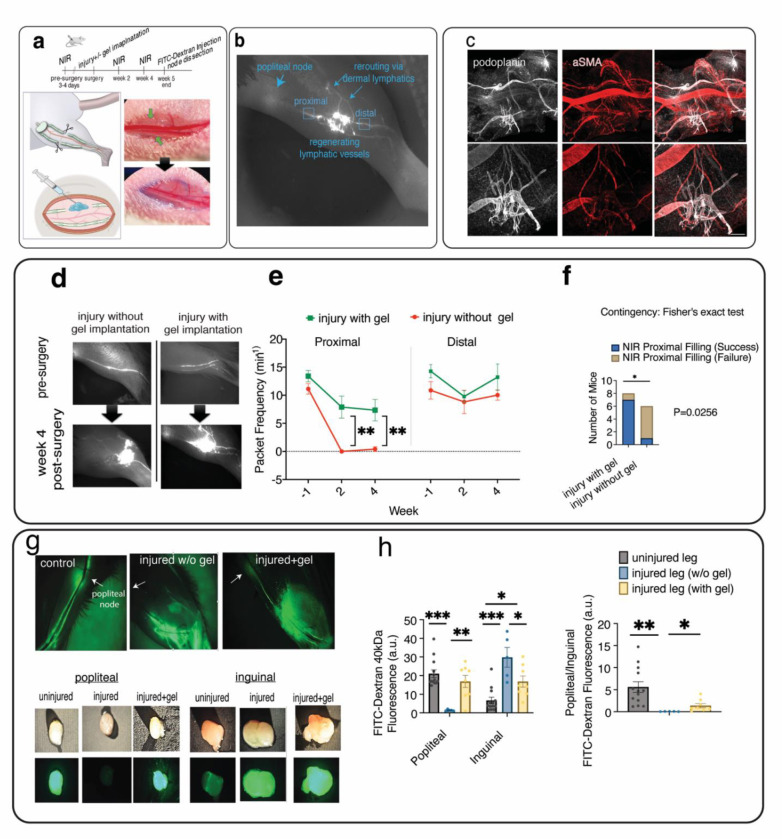
Fibrin gel implantation promotes lymphatic regeneration and restoration of lymphatic transport to the draining lymph node after lymphatic vessel ligation. **a)** Schematic representation of lymphatic surgery where both saphenous lymphatic vessels were surgically ligated, and fibrin gel was implanted. **b)** A representative NIR imaging of mouse hindlimb at four weeks after gel implantation showing reconnection of injured lymphatic vessels as well as rerouting of lymphatic flow to the dermal lymphatic vessels. **c)** Representative confocal imaging showing lymphatic sprouts form from injured collecting vessels following gel implantation to reconnect injured vessel. The image was taken 4 weeks post-injury, where PDPN indicates LECs and αSMA indicates both muscle cells from the saphenous vein and collecting lymphatic vessel. The scale bar indicates 200μm. **d)** Representative longitudinal imaging of lymphatic pumping was captured by a near-infrared imaging (NIR) system, showing popliteal collecting lymphatic vessels before lymphatic surgery and two weeks after injury with and without fibrin gel implantation. **e)** Functional NIR imaging was used to evaluate the restoration of lymphatic pumping in mice that had undergone collecting vessel injury with or without gel implantation (n=6 for injury, n=8 for injury plus gel). Packet frequency, or the number of packet pulsations, was quantified at locations distal and proximal to the site of lymphatic injury. **f)** At 4 weeks post-injury, mice with gel implantation are more likely than mice without gel implantation to have the filling of collecting lymphatic vessels proximal to the injury restored (two-sided Fisher’ exact test p<0.05) **g)** Representative images of 40kDa-FITC uptake into the popliteal and inguinal lymph nodes following lymphatic injury with/without gel implantation after 3 hours. **h)** Quantification of 40kDa-FITC accumulation in the popliteal and inguinal lymph nodes after lymphatic injury with and without gel implantation shows the lymph uptake to the popliteal draining node is restored following gel implantation. After injecting 10ul of 40kDa-FITC dye into the paw, lymph nodes were harvested after 3 hours, and the fluorescence was measured with a fluorometer. Both the injured (with or without gel) and uninjured (control) legs of mouse were analyzed. Unpaired t-test was used to test for significant differences between the NIR metrics of the injured and gel implantation groups at each time (*p < 0.05, **p < 0.01). ANOVA with Tukey post hoc test was used to test the significant difference in FITC uptake (*p < 0.05, **p < 0.01, **p < 0.001). All data were presented as mean (±s.e.m.).

To investigate whether fibrin scaffolding promotes lymphatic regeneration following lymphatic vessel injury, we established a novel mouse model of lymphatic vessel injury in the hindlimb where 1–2 mm sections of the saphenous collecting lymphatic vessels were surgically resected without damaging the adjacent blood vessels (Figure 2a, Supplementary Figure S1, **Video S2**). Fibrin gel was added to the injury site and allowed to polymerize *in situ* (**Supplementary Video S3**). A representative NIR image shows reconnection of injured lymphatic vessels and lymph rerouting to the dermal lymphatics (Figure 2b). Confocal imaging reveals lymphatic vessel sprouting at the site of vessel injury, as indicated by PDPN staining of collecting lymphatic vessels and αSMA staining of both lymphatic and blood vessel muscle cells (Figure 2c, Supplementary Figure S2).

Representative longitudinal NIR imaging indicates that damaging collecting lymphatic vessels without gel implantation results in the loss of lymph transport to the draining lymph node, suggesting a lack of sufficient regeneration and repair following the damage Figure 2d). By contrast, fibrin gel implantation restores lymphatic transport at the site of injury as early as two weeks post-gel implantation (Figure 2d). In mice with injured lymphatic vessels, lymphatic vessel pumping, assessed by the frequency of vessel contractions proximal to the injury site (proximal packet frequency) was significantly higher in the gel implantation group (average frequency = 7.89±1.96min^−1^ at week 2 and 7.34±1.90 min^−1^ at week 4) than the group with no gel (0±0 min^−1^ at week 2, and 0.41±0.41 min^−1^ at week 4). This suggests that gel implantation promotes lymphatic regeneration and restores pumping proximal to the injury site (Figure 2e, Supplementary Figure S3). However, gel implantation had no significant effect on the frequency of vessel contractions distal to the damaged site (distal packet frequency). NIR imaging from 4 weeks post-injury shows that animals with gel implantation are more likely to have their lymphatic flow restored and gain proximal filling (filling of lymphatic vessels proximal to the injury site) than mice without gel implantation (p = 0.0256, Fisher’s Exact test; Figure 2f).

Next, we compared the lymph drainage between the control leg (contralateral leg with uninjured saphenous lymphatic vessels), the injured leg without gel implantation, and the injured leg with gel implantation by quantifying the accumulation of FITC-Dextran in the popliteal draining node and the inguinal lymph node, which is the next draining node that lymph could reach from the injection site in the mouse paw (Figure 2g). In the uninjured leg, the ratio of FITC fluorescence in the popliteal node to the inguinal node was ~5.6, suggesting the popliteal draining node has more than five times the amount of dye accumulation when compared to the inguinal node in the uninjured leg (Figure 2h). After injury, this ratio declines by almost 100 fold (from 5.60±1.19 to 0.054±0.01), implying that injuring collecting lymphatic vessels impedes lymph transport to the draining popliteal node (Figure 2h). Instead, the value of fluorescence in the inguinal node significantly increased ~4.3 folds, from 6.68±1.73 (a.u.) to 28.87±4.42 (a.u.), suggesting compensation by the collateral inguinal lymphatic route after saphenous lymphatic vessel injury. For the injured + gel group, the popliteal to inguinal fluorescence ratio is approximately one, corresponding to the average values of 16.89±3.24 (a.u.) and 16.81±2.99 (a.u.) for popliteal and inguinal nodes, respectively, suggesting that popliteal and inguinal routes equally contribute to lymph drainage after gel implantation. Overall, these results indicate fibrin gel implantation restores impaired lymphatic transport to draining lymph nodes following injury, with some redistribution of lymph between the inguinal and popliteal lymph nodes. In addition, compensation by alternative lymphatic routes facilitates lymph transport following collecting vessel injury.

### Macrophages infiltrate the tissue following lymphatic injury while local depletion of macrophages abrogates lymphatic regeneration after fibrin gel implantation.

**Figure 3.**
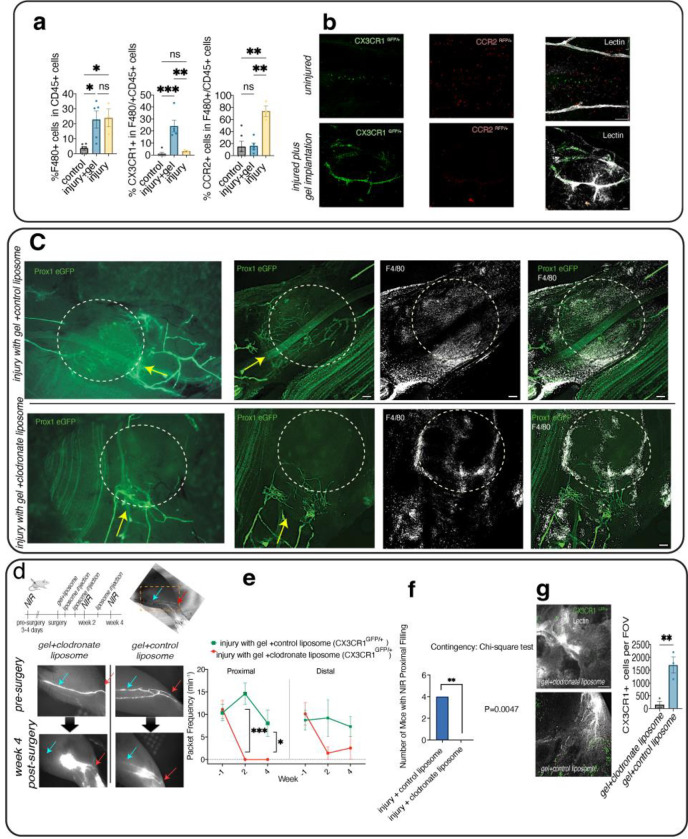
Fibrin gel implantation following lymphatic injury results in macrophage recruitment, while macrophage depletion abolishes lymphatic regeneration after fibrin gel implantation. **a)** Percentage of macrophage infiltration on day 10 after lymphatic injury in the absence and presence of fibrin gel measured by cytometry. **b)** Representative confocal images of the tissue surrounding uninjured and injured collecting lymphatic vessels with gel implantation. The data show that two weeks post-gel implantation, CX3CR1(+) and CCR2(+) cells accumulate around the regenerating vessel, where lectin-647 (white) indicates saphenous lymphatic vessels (see also Supplementary Figure S5). **c)** Representative epifluorescence and confocal images of mouse saphenous tissue treated with control and clodronate liposomes after injury showing clodronate liposomes abolished lymphatic endothelial sprouting (PROX1-GFP+) within the fibrin gel. Mice treated with control liposomes exhibit lymphatic sprouting and reconnection within the gel. The fibrin gel was loaded with 10% v/v clodronate or control liposomes prior to polymerization in vivo. The white circle indicates the approximate injury site, and the white arrows show the direction of lymphatic flow. **d)** Longitudinal near-infrared imaging was used to assess lymphatic pumping and regeneration after macrophage depletion by clodronate liposomes (n = 4) and control liposomes (n = 4) using CX3CR1GFP/+ mice. Representative NIR images of the popliteal collecting lymphatic vessels before surgery, two and four weeks after fibrin gel implantation. The red and blue arrows indicate lymphatics distal and proximal to the injury site, respectively. **e)** Analysis of functional NIR imaging reveals a lack of lymphatic regeneration and loss of lymphatic pumping (packet frequency) after macrophage depletion by clodronate liposomes proximal to the injury site. **f)** After gel implantation and local macrophage depletion via clodronate liposomes in CX3CR1 GFP/+ mice, injured collecting lymphatic vessels did not adequately regenerate to re-connect the injured vessels (failure of proximal filling). In contrast, CX3CR1 GFP/+ mice treated with control liposomes successfully reconnected injured vessels (success of proximal filling two-sided Fisher’s Exact test p<0.05). **g)** Confocal imaging was used to evaluate the depletion success in CX3CR1^GFP/+^ mice. Quantification of CX3CR1+ cells shows that the number of cells significantly decreases following depletion by clodronate liposomes (n=3). Statistical significance differences between presurgical values and values on days 14, and 28 following injury were tested via ANOVA with Tukey’s post hoc tests (*p < 0.05, **p < 0.01). Unpaired t-test was used to test for significant differences between the control liposomes and clodronate liposome groups at each time (*p < 0.05, ** p<0.01).

To investigate whether macrophages infiltrate into the fibrin gel, we performed flow cytometry on the tissue surrounding the saphenous collecting lymphatic vessels 10 days after injury. Flow cytometry revealed a significant increase in the percentage of F4/80(+) macrophages in the tissue after injury to the collecting lymphatic vessels (Figure 3a, Supplementary Figure S4). The data indicate the presence of two distinct macrophage phenotypes after lymphatic injury, with CCR2(+) macrophages infiltrating tissue in the absence of fibrin gel and CX3CR1(+) patrolling macrophages increasing with gel implantation (Figures 3a, b, Supplementary Figure S5).

To test whether macrophage infiltrates contribute to lymphatic regeneration and pumping restoration, we depleted macrophages locally via clodronate liposomes in mice with collecting lymphatic vessel injury and fibrin gels (Figure 3c). We compared functional NIR pumping metrics in mice treated with clodronate liposomes to those treated with control liposomes (Figure 3d, n=4 each group). NIR imaging revealed that clodronate treatment results in abrogation of lymphatic regeneration and the lack of restoration of lymphatic contractions (Figure 3e). Lymphatic contractions were not observed proximal to the injury site following treatment by clodronate liposomes (proximal contraction frequency = 11.42±2.28 min^−1^ pre-surgery, 0±0 min^−1^ on days 14 and 28 following injury, gel implantation and macrophage depletion), indicating a lack of lymphatic regeneration after treatment with clodronate liposomes. In addition, distal packet frequency declined by almost 10 fold (from 13.46±1.58 min^−1^ pre-surgery to 1.4±1.4 min^−1^ on day 14, and 1.32±1.32 min^−1^ on day 28). In the control liposome group, the pre-surgical values of packet frequency at the proximal injury site were not significantly different compared to the values on days 14 and 28 (from 9.45±1.8 min^−1^ pre-surgery to 7.92±2.87 min^−1^ on day 28), suggesting lymphatic regeneration was not significantly impaired by treatment with control liposomes.

NIR results from 4 weeks after treatment also show that mice treated with clodronate liposomes are less likely to gain proximal filling than mice without gel implantation (p = 0.0256, Fisher’s Exact Test, Figure 3f). Confocal imaging after week 4 confirmed that macrophage depletion was successful, showing a nearly 10 fold decrease in CX3CR^GFP/+^ cells at the surgical site in mice treated with clodronate liposomes (1707.3±305.4 cells per field of view (FOV) in the control liposomes group vs. 155.3±123 cells in the clodronate liposomes group; n=3 each group and FOV=1.6 mm^2^; Figure 3g).

### Fibrin gel implantation results in increased tissue CCL5 following lymphatic vessel injury and genetic ablation of CCL5 inhibits regeneration.

**Figure 4.**
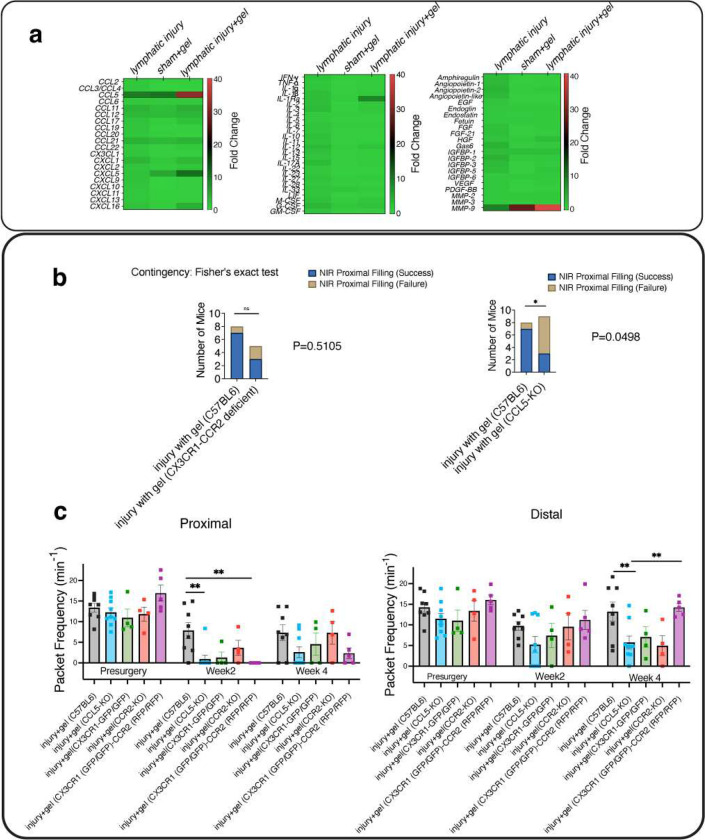
Fibrin gel implantation leads to increased tissue CCL5 after lymphatic vessel injury and loss of CCL5 inhibits the recovery of lymphatic pumping proximal to the injury site. **a)** A comparative analysis of the levels of cytokines and growth factors after gel implantation demonstrating the upregulation of CCL5 and MMP9 (n=3 mice pooled in each group)**. b)** Mice lacking CCL5 (CCL5−/−) cytokine are less likely than control mice to regain filling of vessels proximal to the injury site (proximal filling) subsequent to gel implantation 4-week post-injury (p=0.0498, two-sided Fisher’s Exact Test) while deficiency of CX3CR1 and CCR2 are not associated with the success/failure of proximal filling recovery following gel implantation (p=0.5105, two-sided Fisher’s Exact Test). **c)** A comparison of lymphatic pumping frequencies via NIR technique proximal and distal to the injury site among CCL5-KO, CX3CR1(GFP/GFP), CCR2(−/−), and double deficient CX3CR1(GFP/GFP)-CCR2(RFP/RFP) mice following gel implantation. To test the association between categorical variables (success/ failure of proximal filling after gel implantation), the two-sided Fisher’s Exact test was used (p < 0.05). One-way ANOVA analysis with the Tukey post hoc test was used to test the significant difference between different groups after gel implantation (*p < 0.05, **p < 0.01, ***p < 0.001). All data were presented as mean (±s.e.m.).

To determine which cytokines were upregulated as a result of lymphatic injury and gel implantation, we conducted a semi-quantitative cytokine array analysis on day 10 following lymphatic surgery and fibrin gel implantation. Interestingly, the array suggested that CCL5, but not CCL2 or CX3CL1, increased 31.6-fold after injury plus gel implantation, compared to a 9.6-fold increase for injury and a 10.3-fold increase for gel plus sham surgery, when normalized to the sham surgery group (Figure 4a, Supplementary Figure S6). In addition, we also measured an increase in the matrix metalloproteinase MMP9 in mice with gel implantation after lymphatic injury (Figure 4a, Supplementary Figure S6). It is known that CCL5 functions as a ligand for CCR1, CCR3, and CCR5 to recruit macrophages and T cells to the site of injury (42, 43). Thus, we set out to determine if genetic deletion of CCL5 inhibited lymphatic regeneration and the subsequent recovery of lymphatic pumping proximal to the injury site. In addition, the expression of CCR2 and CX3CR1 by infiltrating cells prompted us to determine if a deficiency in CCR2 or CX3CR1 impairs lymphatic regeneration.

According to NIR data from week 4 after injury (Figure 4b), mice lacking CCL5 are less likely than control mice to recover their proximal filling (filling of lymphatic vessels proximal to the injury site) following gel implantation (p=0.0498, Fisher’s Exact test). However, repeating the experiments in double mutant *Cx3cr1* and *Ccr2* mouse model, CCR2^RFP/RFP^CX3CR1 ^GPP/GFP^ mice, showed the number of mice having their collecting vessel, proximal to the injury site, filled with NIR dye, was not significantly different when compared to control mice. This suggests that deficiency in CX3CR1 and CCR2 is not associated with impaired regeneration 4 weeks after injury and gel implantation, (p=0.5105, Fisher’s Exact Test). Interestingly, representative confocal imaging shows that deficiency in CCR2 or CX3CR1 does not prevent infiltration of cells expressing CX3CR1 and CCR2 (Supplementary Figure S7), suggesting these receptors are not involved in the trafficking of macrophages to the site of injury. Further, NIR imaging at week 4 post-surgery shows no significant difference in proximal pumping frequency between mice lacking CCR2 (7.32±2.71 min^−1^ for CCR2^−/−^ mice, n=4), CX3CR1 (6.09±3.08 min^−1^ for CX3CR1 ^GPP/GFP^, n=4), CCL5 (2.61±1.33 min^−1^ CCL5−/− mice, n=9) the double deficient CX3CR1 and CCR2 (2.35±1.29 min^−1^ CCR2^RFP/RFP^CX3CR1 ^GPP/GFP^, n=5) and wild-type mice (7.34±1.90 min^−1^ C57BL6, n=8) (Figure 4c).

## Discussion

Disruption of lymphatic pathways due to cancer surgery, radiation therapy, traumatic injuries, or infection are common causes of secondary lymphedema (44). The notion is that lymphedema is caused by an inadequate lymphatic regeneration response and subsequent pumping dysfunction after cancer surgery and therapy, which manifests as lymph accumulation in the body’s soft tissues. (79). Our major finding is that implanting an optimized fibrin hydrogel after lymphatic vessel injury led to lymphatic regeneration and restoration of lymphatic flow as early as two weeks post-gel implantation (80). The results also revealed that LECs sprouted from injured collecting lymphatic vessels to reconnect injured vessels. Furthermore, compensatory lymphatic routes redirect lymph transport to other nodes after the injury of the primary lymph drainage route. In addition, a collateralization response reroutes lymph to dermal lymphatics to compensate for reduced collecting lymphatic vessel transport after injury.

Fibrin plays a major role as the first scaffold that cells encounter after injury, aiding in the adhesion of a variety of immune cells such as platelets, neutrophils, and macrophages, which facilitate tissue regeneration and repair (45, 46). Several studies have also used collagen and fibrin hydrogels as scaffolds to study lymphatic sprouting (28, 33). Helm et al. have shown that a fibrin-only matrix provides the optimal matrix for LEC organization *in vitro*, compared to a fibrin-collagen mixture and collagen-only matrices (47). In addition, seeding LECs with adipose-derived stem cells (ADSCs) in a fibrin scaffold has been shown to induce the formation of a lymphatic network, suggesting that ADSCs and fibrin hydrogel have favorable effects on LEC differentiation (32). In a porcine lymphedema model, implanting nano-fibrillar collagen scaffolds has been reported to increase lymphatic vessel density (31). Consistent with earlier studies, our findings indicate that the fibrin scaffold effectively supports lymphatic regeneration. When cultured on soft matrix, LECs are known to express more genes associated with cell migration (48). Our data also show sprouting responses tend to decrease at higher fibrinogen concentrations, with the optimal sprouting response occurring at a concentration of 9 mg/mL, corresponding to a storage modulus (G’) of 555 Pa. *In* vitro sprouting of collecting lymphatic vessels showed the presence of PDPN (+) cells along with partial coverage by αSMA (+) cells, indicating that sprouts contain both LECs and LMCs. Immunostaining of saphenous tissue, four weeks after collecting vessel injury and gel implantation, also confirmed sprouts at the site of collecting lymphatic vessel injury, expressing PDPN with partial αSMA coverage.

We also found that two distinct macrophage phenotypes infiltrate the tissue after collecting lymphatic vessel injury: i) CCR2+ macrophages that increase in the tissue after injury without gel implantation; ii) CX3CR1+ patrolling macrophages that infiltrate tissue after fibrin gel implantation. Our findings suggest that macrophages expressing CX3CR1(+) infiltrate fibrin gel and accumulate in proximity to regenerating lymphatic vessels. In general, monocytes and monocyte-derived macrophages have been reported to express CCR2 and CX3CR1 receptors, with inflammatory monocytes expressing high levels of LY6C and CCR2 (CX3CR1^low^) and patrolling/reparative monocytes expressing high levels of CX3CR1 (CCR2^low^ LY6C^low^) (49). Earlier investigations have also reported that CCR2(+) and double-positive CCR2 and CX3CR1 macrophages infiltrate fibrin implants and contribute to fibrin endocytosis and clearance (50). In addition, tissue-resident macrophages expressing CX3CR1 have been shown to regulate lymphatic vessel growth in cardiac lymphatics via direct interaction with LECs (51). Similarly, embryos lacking macrophages have been shown to develop malformed lymphatics, implying macrophages play an important role in lymphatic morphogenesis (52). Interestingly, we found that the deficiency in CCR2 or CX3CR1 function did not significantly inhibit macrophage infiltration and lymphatic regeneration into the fibrin gels after lymphatic injury. Consistent with this observation, previous research has suggested LY6C^low^ patrolling/reparative monocytes rely on CCR5, but not CCR2 or CX3CR1, to migrate to the injury site (53). CCL5, expressed by endothelial cells and activated leukocytes, is a potent chemoattractant for monocytes and lymphocytes acting via CCR1 and CCR5 receptors (42, 43). Upon stimulation, LECs and LMCs have been shown to secrete CCL5 chemokine (54, 55). Mice lacking CCL5 have also been reported to exhibit impaired monocyte recruitment and T cell function, as well as significantly reduced swelling in the delayed-type hypersensitivity assay (56). Notably, we found CCL5, but not CCL2, CCL3, or CX3CL1, is markedly elevated with the implantation of fibrin gel after lymphatic injury. We also observed that genetic ablation of CCL5 cytokine impairs the lymphatic regeneration process. In this regard, CCL5 may contribute to lymphatic regeneration in several ways. Monocytes have been demonstrated to increase MMP-9 production in response to CCL5 stimulation (57). MMP-9 cleaves ECM-bound growth factors and degrades type IV collagen in the basement membrane, allowing increased endothelial migration and sprouting (58). We also found that MMP9 was highly elevated after fibrin gel implantation, suggesting that CCL5 could be involved in MMP-9 upregulation (Figure 4a, Figure S6). Aside from being a chemokine for monocytes, CCL5 is also known to recruit endothelial progenitor cells—c-Kit^+^Tie-2^+^ cells expressing CCR5—from the circulation to promote neovascularization at the site of injury (59). A subset of mesenteric LECs has also been reported to originate from cKit+ hemogenic endothelial cells (60). In addition, CCL5 may play a role in VEGF-C production, as CCL5 has been reported to induce VEGF-C production in human chondrosarcoma cells (61).

One limitation of our study is that the injury model doesn’t induce edema, and thus we are limited in our ability to study the benefit of gel implantation in preventing lymphedema. In humans, lymphedema occurs in a delayed fashion months to years following lymphatic damage caused by cancer surgery and treatment (62). In contrast, swelling caused by damage to lymphatic vessels and skin in mice resolves on its own if given enough time (41, 63). In agreement with previous studies, we also did not observe persistent swelling following popliteal lymphatic damage. One potential explanation is that the mouse models cannot fully recapitulate lymphatic pathophysiology, such as the effect of gravitational dependence on lymphedema progression. Further, a recent report suggests that injury in sheep hindlimbs doesn’t lead to lymphedema and that collecting lymphatic vessels may spontaneously regenerate following injury (64). This is in contrast to our finding that, in the absence of scaffolding, collecting lymphatic vessels do not adequately regenerate to re-connect injured lymphatic vessels. This disparity could be due to the severity of the injury to the hindlimb of a sheep, where the lymphatic excision was relatively small. Moreover, differences in the preexisting lymphatic network in the hindlimbs of mice and sheep, which can influence how lymph transverses to the preexisting network of lymphatic pre-collectors, may account for the observed disparity.

In summary, our findings provide insight into how fibrin scaffolding promotes lymphatic regeneration, resulting in the reconnection of the injured lymphatic vasculature and the re-establishment of lymphatic pumping to the draining node. Further, our data provide evidence that fibrin scaffolding recruits macrophages in a CCL5-dependent manner and that the CCR5+ macrophages are able to contribute to lymphatic regeneration. Include a Discussion that summarizes (but does not merely repeat) your conclusions and elaborates on their implications. There should be a paragraph outlining the limitations of your results and interpretation, as well as a discussion of the steps that need to be taken for the findings to be applied. Please avoid claims of priority. Do not include a “Conclusion” heading.

## Materials and Methods

### Mice.

C57BL/6 mice, and αSMA-DsRed (C57BL/6) were obtained from the Cox-7 animal facility operated by the Edwin L. Steele Laboratories, Department of Radiation Oncology at MGH. PROX1-eGFP (kind gift of Taija Mäkinen, Uppsala University and Young-Kwon Hong, University of Southern California), CX3CR1^GFP/+^ and CX3CR1^GFP/+^CCR2^RFP/+^ reporter mice (C57BL/6, JAX), double mutant CX3CR1^GFP/RFP^CCR2^RFP/RFP^ mice (C57BL/6, JAX), CCL5-KO mice (C57BL/6, JAX), were housed at MGH Center for Comparative Medicine facilities. Female and male mice (8–20-week-old) were used in this study. Animal protocols were approved by the Institutional Animal Care and Use Committees (IACUC) at MGH, and all facilities are accredited by the Association for Assessment and Accreditation of Laboratory Animal Care International (AAALAC).

### Lymphatic injury surgical procedure.

Mice were anesthetized with administration of ketamine (10 mg/kg) and xylazine (100 mg/kg). To enhance the visibility of lymphatic vessels, 5 *μ*L of Evans Blue (0.1% diluted in PBS) was injected into the mouse footpad. Fur was removed by shaving and applying a depilatory cream. To gain access to two saphenous lymphatic vessels—the afferent collecting lymphatic vessels to the popliteal lymph node—that run along the saphenous vein, a skin incision distal to the knee in the mouse hindlimb was made using a scalpel. A small segment of the popliteal lymphatic vessel (1–2 mm) was excised using a microsurgical scissor and under a stereomicroscope. Care was taken to prevent damage to the saphenous vein and mice with bleeding were excluded. For the gel implantation studies, ~10 *μ*L of unpolymerized fibrin gel (a mixture of 9mg/ml fibrinogen and 2U/mL thrombin diluted in Opti-MEM) was added to the injury site by pipette. Once the procedure was completed, the incision site was sutured (number 5–0, Ethicon). Buprenorphine (0.05–0.1 mg/kg) was used post-operatively as an analgesic.

### 3D lymphatic vessel culture in fibrin gel.

Segments of collecting lymphatic vessels were embedded in a fibrin gel in a 48-well plate. Briefly, fibrinogen (Sigma, cat#F8630) was diluted in Opti-MEM (Gibco, cat#31985062) to yield the desired concentration. The solution was incubated for at least one hour at 37 °C to dissolve, and then it was filtered (0.22 μm) to remove unpolymerized fibrinogen. Aliquots of fibrinogen solutions (30 mg/mL, 18mg/mL, and 6mg/mL) were placed on ice. Thrombin (Sigma cat#T4648) at 4U/mL was added to each fibrinogen aliquot (1:1 ratio) to begin the polymerization of fibrinogen into fibrin strands immediately. Segments of collecting lymphatic vessels were encapsulated between two 200uL fibrin layers in a 48-well plate. After polymerization, 800uL of MCDB 131 Medium (Gibco, cat# 10372019), supplemented with 4% Ultroser^™^ G serum substitute, 1% hydrocortisone (STEMCELL Technologies, cat# 74142), 1% L-Glutamine (Gibco^™^), 1% Penicillin-Streptomycin (Gibco^™^), was used to culture segments under 5%O_2_ and 5%CO_2_ for six days (28).

### Fibrin rheological measurement.

Fibrin viscoelastic properties were measured using an oscillatory shear rheometer (AR-G2, TA Instruments, DE) as described previously (34, 35). In brief, a 40-mm diameter parallel plate was utilized, and the plate temperature was controlled at 37 °C throughout the measurement. The unpolymerized fibrin precursor solution was pipetted onto the bottom plate of the rheometer before the top plate was lowered to a gap of 500 μm such that the solution completely filled the space between the two plates. With a moisture trap placed around the plates, the fibrin hydrogel was allowed to polymerize for 15 minutes. After polymerization, a frequency sweep test was performed with 1% strain over a frequency range of 0.1–100 Hz. Three replicates were tested for each gel concentration. Measurements at 1 Hz were used to compare storage modulus (G’) values at different gel concentrations.

### FITC-Dextran uptake experiment.

10μL of 1% FITC-Dextran (40,000 MW, Sigma) in PBS was injected into the dorsal aspect of the mouse paw. Popliteal and inguinal lymph nodes were harvested 3 hours after the injection. Each node was homogenized in 1mL of PBS using a tissue homogenizer. 300uL of each sample was transferred in a 96-well plate, and fluorescence was measured using a Fluorescent Ascent^™^ Fluorometers (Thermo Scientific).

### Near-infrared imaging.

Lymphatic near-infrared imaging (NIR) was used to assess lymphatic pumping and regeneration in vivo. Mice were anesthetized with ketamine/xylene. Hair was removed using a depilatory cream, and 5μL of Dextran (10K), Flamma^®^ 774 (BioActs), was injected intradermally into the dorsal aspect of the mouse paw. To image the popliteal afferent lymphatic vessels, near-infrared imaging was performed using a previously described NIR setup (36). Briefly, the imaging system is composed of a ×6.5 Zoom lens (Navitar), and a Prosilica GT2750 camera (Allied Vision Technology) with an ICG-B emission filter (832/37, Semrock). A custom-built ring light source was used to excite the dye using 760 nm high-power laser-emitting diodes (Marubeni) filtered through a 775/50 bandpass filter (Chroma). A custom-written MATLAB code was used to analyze images.

### Macrophage depletion experiment.

Clodronate liposomes and control liposomes were purchased from Liposoma BV. Fibrin gel was prepared by mixing a 1:1 ratio of fibrinogen (18mg/mL) and thrombin solution (4U/mL) as described previously. After ligating afferent popliteal lymphatic vessels in CX3CR1^GFP/+^ mice, liposomes were immediately added to fibrin gel (10% v/v) before polymerization and 10μL of fibrin gel loaded with clodronate liposomes was implanted at the injury site. Subcutaneous injection of 10μL of clodronate liposomes at the injury site was repeated at 3, 7, 14, 21 days after gel implantation. In control experiments, control liposome (10% v/v) was added in the fibrin gel and subcutaneous injections of control liposome were repeated on days 3, 7, 14, 21 after surgery and gel implantation. NIR functional lymphatic imaging was performed ~3–4 days before surgery and at 2- and 4-weeks post-surgery. Finally, popliteal tissues from CX3CR1^GFP/+^ mice were harvested, and confocal imaging was performed to confirm macrophage depletion by clodronate liposomes.

### Single cell suspension and flow cytometry.

To prepare a single-cell suspension, saphenous tissue, including popliteal lymphatic vessels afferent to the popliteal lymph node, were dissected from individual mice. Harvested tissues were placed on ice-cold DMEM 1X (Corning, with 4.5 g/L glucose, L-glutamine & sodium pyruvate), minced with surgical scalpel and fine forceps. Minced tissues were transferred into 5mL test tubes containing 2 mL of freshly made enzyme mix containing (0.8 mg/mL Dispase II (Sigma), 0.2 mg/mL Collagenase P (Roche), and 0.01mg/mL DNase (Roche). Samples were incubated at 37° C with gentle rocking for 30 minutes. Following incubation with enzyme mix, the digested tissue was aspirated using a 1-mL pipette, 3 mL of DMEM 1X was added to the mixture, which was then filtered through a 70 μm nylon mesh. The mixture was centrifuged (1500 rpm, 5 min at 4 °C), the supernatant was decanted, and the cell pellet was resuspended in 200μL of ice-cold FACS buffer (1% BSA in PBS). The cells were blocked using anti-CD16/32 antibodies. Cell surface markers were stained for 20 minutes on ice (see Table S1 for a list of antibodies). Flow cytometry was performed with FACSAria (B.D. Biosciences). Data analysis was performed via FlowJo (Tree Star).

### Proteome profiler cytokine array.

Proteome profiler cytokine array (R&D systems, Minneapolis, MN) was used to screen relative levels of inflammatory cytokines following lymphatic injury and gel implantation according to the manufacturer’s protocol. There were four groups, as follows (i) sham injury: in the sham group, a skin incision was made without injuring lymphatic vessels; (ii) lymphatic injury: in the injury group, a section of afferent lymphatic vessel to the popliteal lymph node was surgically removed, but the gel was not implanted; (iii) sham injury plus gel, a skin incision was made with a scalpel, gel was implanted at the site of the injury without injuring lymphatic vessels; ( iv) injury plus gel, a section of afferent lymphatic vessel to the popliteal lymph node was surgically removed and the fibrin gel was implanted. After 10 days, tissues were harvested from the saphenous region and homogenized using a handheld homogenizer according to the manufacturer’s protocol. Tissue lysates were used for cytokine arrays, as directed by the manufacturer. Cytokine expression was semi-quantitively determined via densitometric analysis by Protein Array Tool in MATLAB (37). The data were presented as fold-change in comparison to the sham group (see Figure S3 for array images).

### Immunostaining.

Lymphatic sprouts from *in vitro* culture were fixed for 30 minutes in 4% PFA (vol/vol) at room temperature. The sprouts were washed three times with PBS and placed in 10% donkey serum and 0.5% Triton X-100 in PBS for 1 hour at room temperature. After incubation with the podoplanin (PDPN) antibody overnight at 4 °C, segments were washed with PBS and incubated with the secondary antibody, or αSMA-Cy3, overnight at 4 °C. After washing with PBS three times, the segments were placed on the microscope slide to perform imaging. For staining with lectin, segments were incubated with diluted lectin (1:100) over night after fixation. For wholemount staining of collecting lymphatic vessels from mice with PDPN, 10 μL of diluted PDPLN in PBS (1:50) and FcBlock (1:500) were injected into the mouse footpad. After 24 hours, 10 μL of diluted secondary antibody was injected into the mouse foot. The popliteal tissue was then harvested after 1 hour. For lectin wholemount staining, 10 μL of diluted lectin was injected into the mouse footpad and tissue was harvested 1~2 hours after the injection. For αSMA or F4/80 wholemount staining, the popliteal tissue was fixed with 4% PFA for 30 minutes, washed with PBS, and incubated with conjugated antibody overnight at 4°C. After washing, the tissue was placed on a glass slide to visualize lymphatic vessels under a confocal microscope. Please see Supplementary Table 1 for the details of antibodies.

### Software and Statistical analysis.

AngioTools Software and Neurite-J plugin were used to measure various morphometric parameters of lymphatic sprouts (38, 39). In brief, Nurite-J and ImageJ were used for image segmentation. Angiotools was used to quantify morphometric parameters. To analyze confocal images and quantify cells based on the fluorescence staining, the Colocalization Object Counter plugin and FIJI were used (40). A custom-written MATLAB script was used to analyze NIR data and obtain lymphatic pumping metrics (41). Schematic figures were created with BioRender. All statistical analysis was performed in Prism 9. Statistical significance was tested using a one-way ANOVA with the Tukey post hoc test or a two-tailed Student’s t-test, as appropriate. To test if there is a nonrandom association between categorical data (e.g., test if there is an association between gel implantation and the success of regeneration) Fisher’s Exact test was used. The significance was set at P<0.05.

## Supplementary Material

Supplement 1
