# Supplementary material for: Regeneration of collecting lymphatic vessels following injury
Source: Res Sq. 2023 Jul 3:rs.3.rs-3025656. Preprint. [Version 1] doi: 10.21203/rs.3.rs-3025656/v1 (PMC10350186; doi:10.21203/rs.3.rs-3025656/v1)
Supplement: Supplement 1 [file NIHPPRS3025656V1-supplement-1.pdf]

Supplementary Materials

Regeneration of collecting lymphatic vessels following injury

Mohammad S. Razavi *et al*

*Corresponding author. Email: tpadera@steele.mgh.harvard.edu; : munn@steele.mgh.harvard.edu

Figs. S1 to S7
Tables S1
Movies S1 to S6

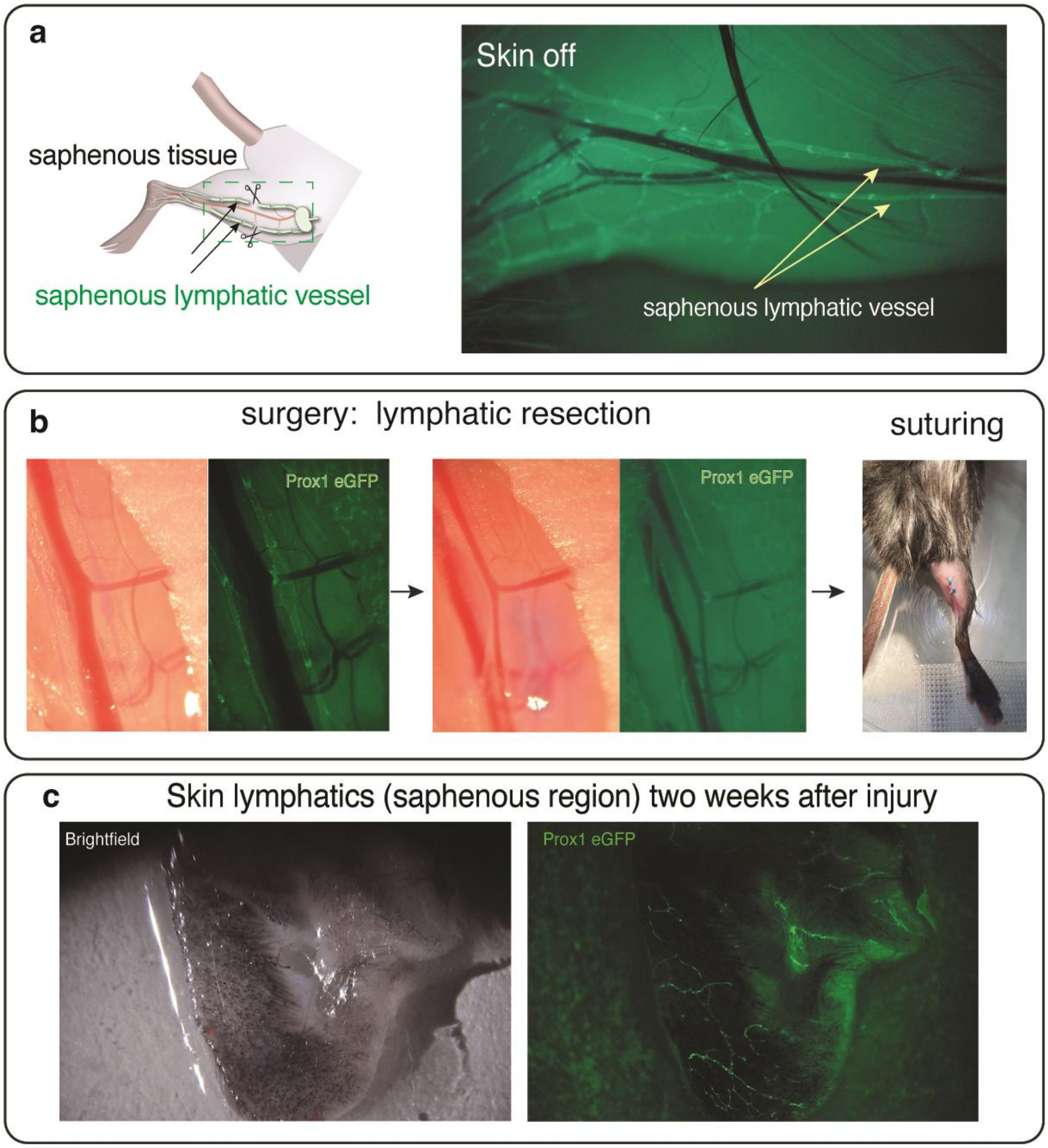

Figure S1 a) A schematic representation of the saphenous region and fluorescent imaging in Prox-1eGFP mice exhibit saphenous lymphatic vessels b) Demonstration of lymphatic vessels prior to the surgery and after the surgery c) The skin of the saphenous region on top of the lymphatic vessels after two weeks post-injury demonstrates the presence of Prox-1-positive lymphatic vessels, suggesting the rerouting of collecting lymphatic vessels to the skin lymphatics after injury.

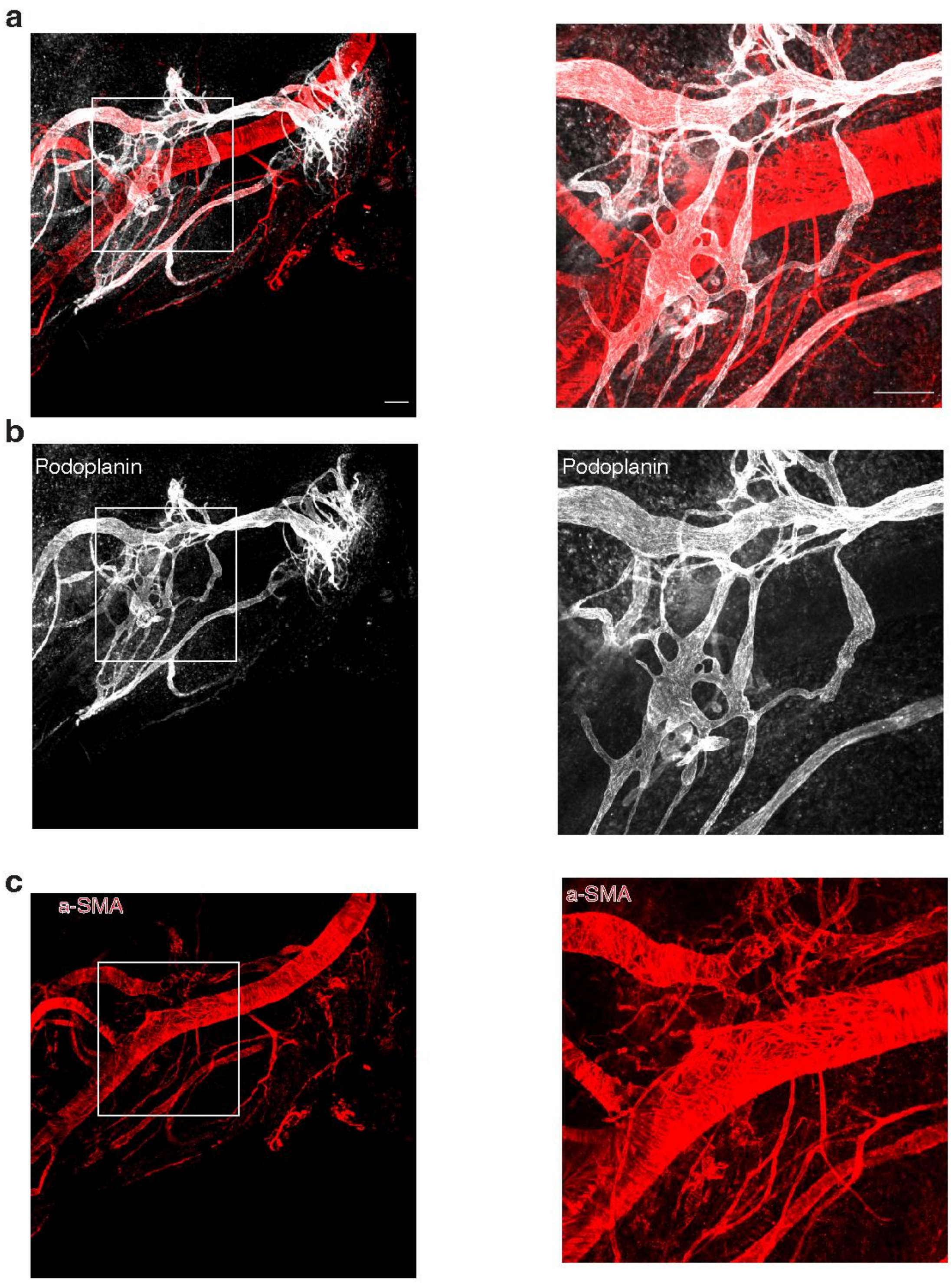

Figure S2 a) Immunostaining shows lymphatic vessel sprouting at the site of vessel injury after 4-weeks post-gel implantation b) PDPN staining indicates LECs from collecting lymphatic vessels and c) αSMA staining shows lymphatic and blood vessel muscle cells. The scale bar indicates 200μm.

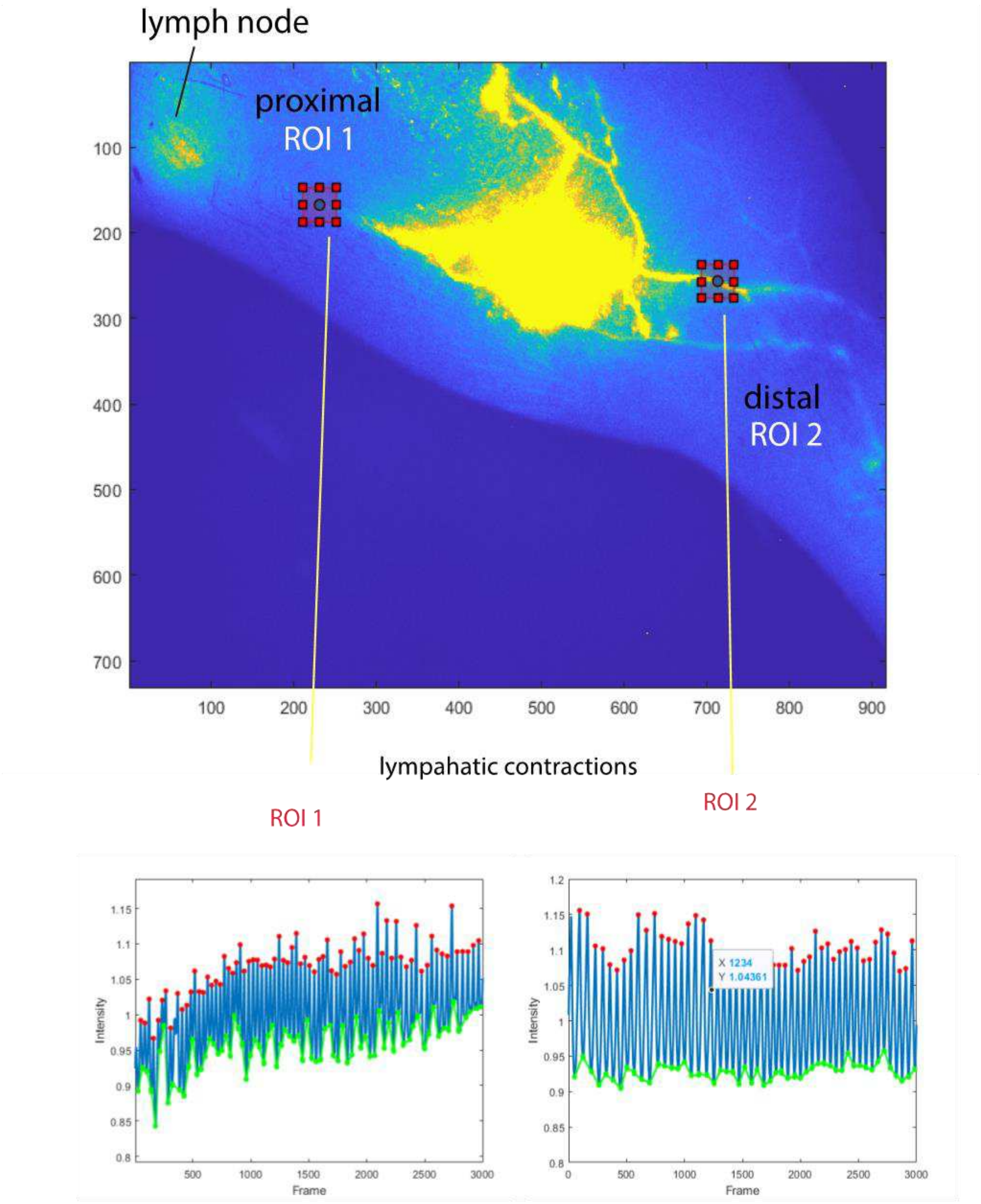

Figure S3 A functional NIR imaging example showing analysis of NIR signal pulsation associated with vessel contraction proximal and distal to the injury site four week after gel implantation. Images were acquired at 10 frames per second. The number of NIR signal peaks associated with vessel diastole was used to quantify contraction frequency.

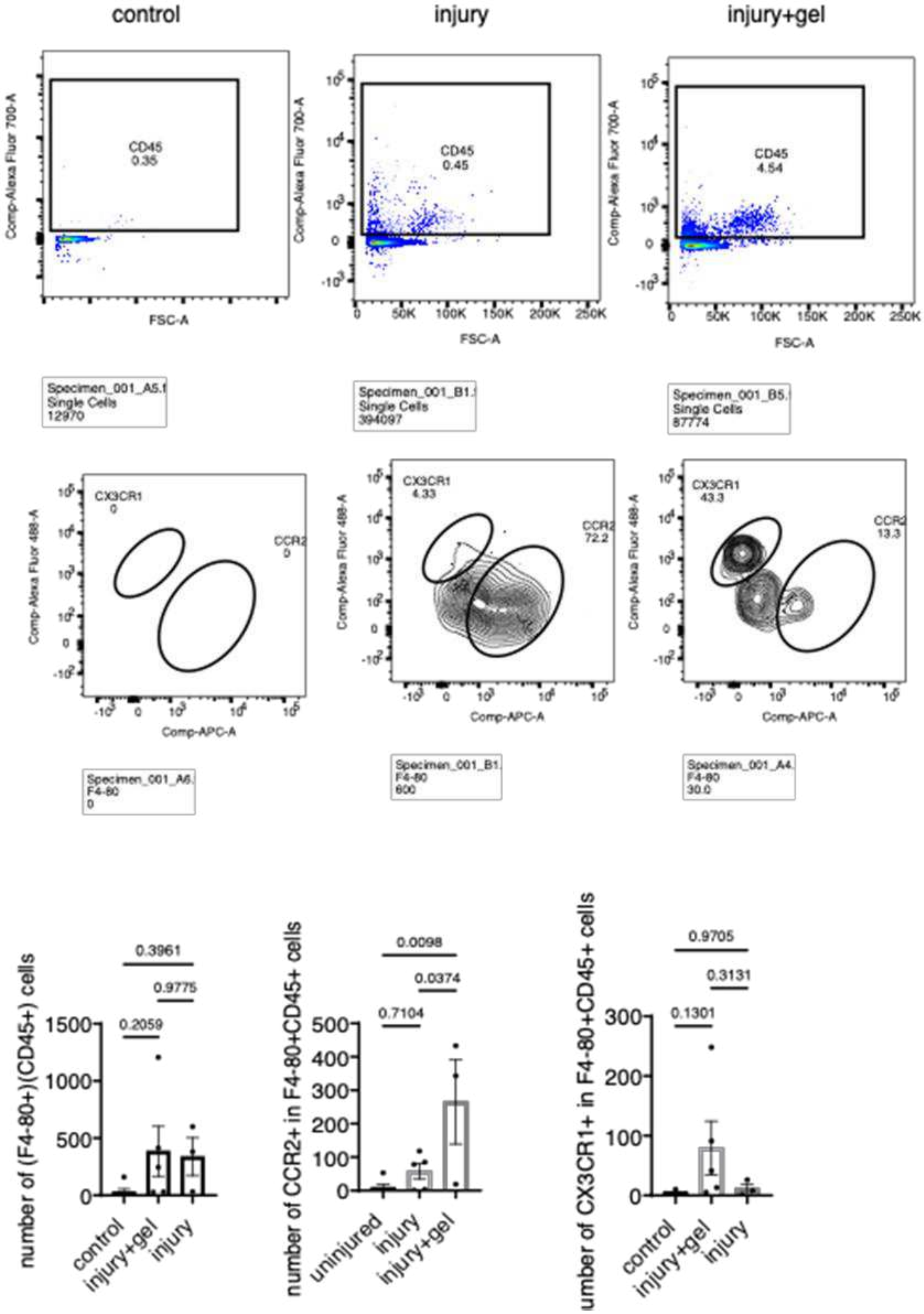

Figure S4 The gating strategy and the results of quantification of macrophage subtypes after collecting vessel injury with or without gel implantation.

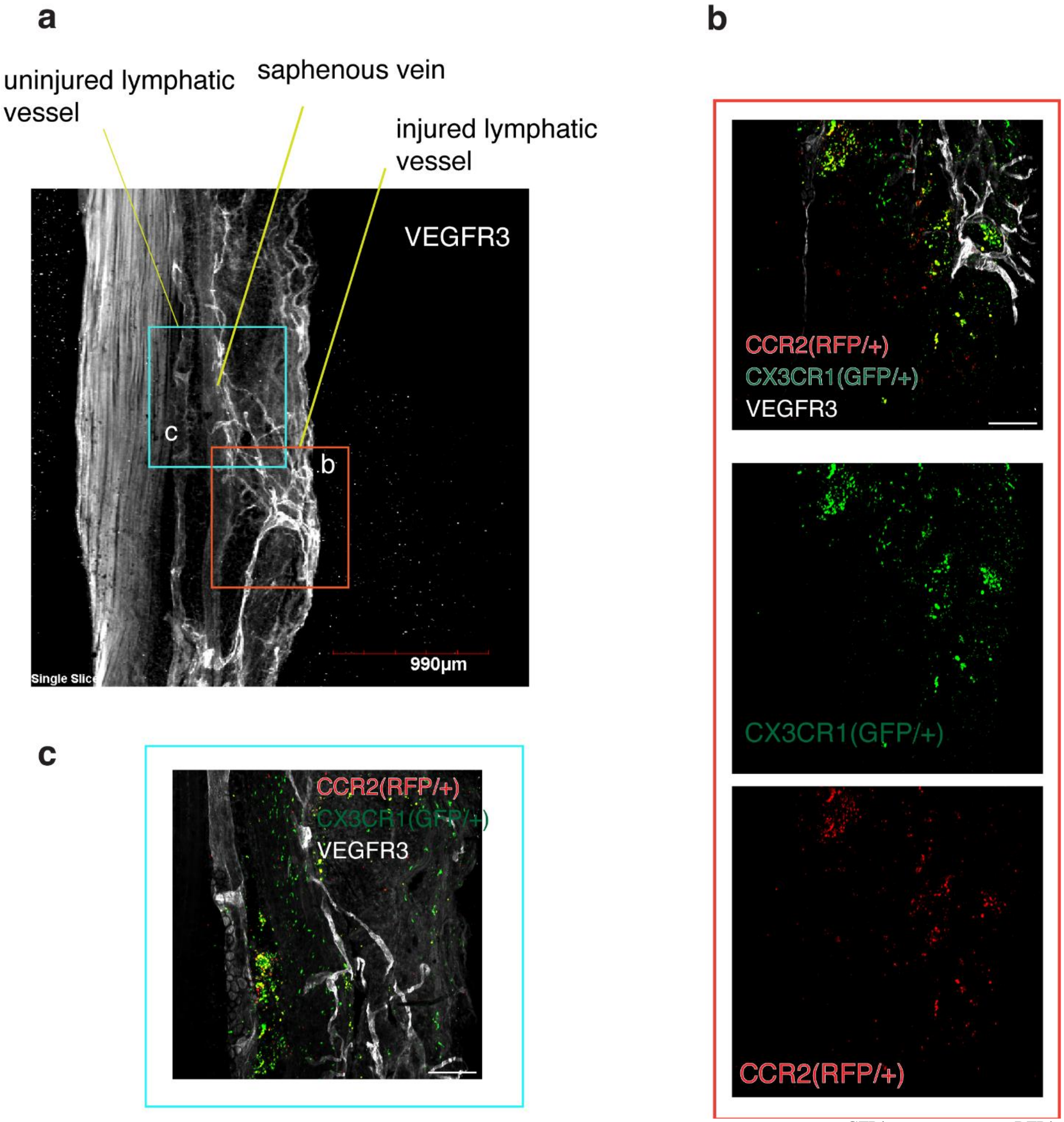

Figure S5 Representative confocal imaging showing infiltration of CX3CR1$^{GFP/+}$ and CCR2$^{RFP/+}$ cells 4 weeks after gel implantation. a) whole-mount tissue staining of saphenous tissue indicates sprouting at the site of collecting lymphatic vessel injury b-c) higher magnification images show infiltration of CX3CR1$^{GFP/+}$ cells and CCR2$^{RFP/+}$ cells around the injury site and lymphatic sprouts.

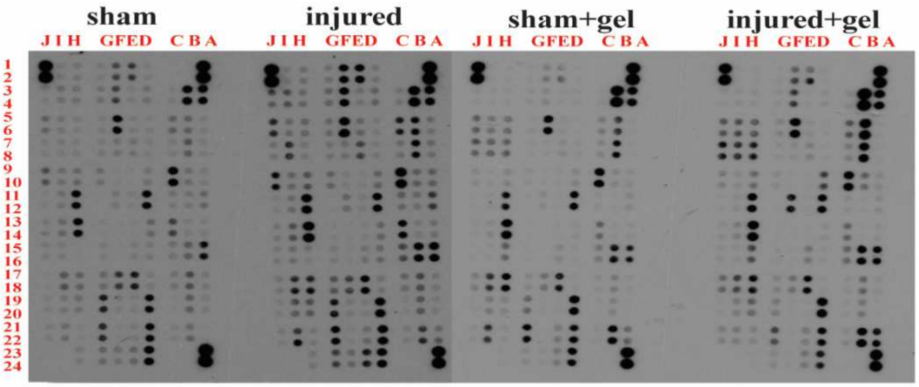

Coordinate	Analyte/Control
A1, A2	Reference Spots
A3, A4	Adiponectin/Acrp30
A5, A6	Amphiregulin
A7, A8	Angiopoietin-1
A9, A10	Angiopoietin-2
A11, A12	Angiopoietin-like 3
A13, A14	BAFF/BLyS/TNFSF13B
A15, A16	C1q R1/CD93
A17, A18	CCL2/JE/MCP-1
A19, A20	CCL3/CCL4/MIP-1α/β
A21, A22	CCL5/RANTES
A23, A24	Reference Spots
B3, B4	CCL6/C10
B5, B6	CCL11/Eotaxin
B7, B8	CCL12/MCP-5
B9, B10	CCL17/TARC
B11, B12	CCL19/MIP-3β
B13, B14	CCL20/MIP-3α
B15, B16	CCL21/6Ckine
B17, B18	CCL22/MDC
B19, B20	CD14
B21, B22	CD40/TNFRSF5
C3, C4	CD160
C5, C6	Chemerin
C7, C8	Chitinase 3-like 1
C9, C10	Coagulation Factor III/Tissue Factor
C11, C12	Complement Component C5/C5a
C13, C14	Complement Factor D
C15, C16	C-Reactive Protein/CRP
C17, C18	CX3CL1/Fractalkine
C19, C20	CXCL1/KC
C21, C22	CXCL2/MIP-2
D1, D2	CXCL9/MIG
D3, D4	CXCL10/IP-10
D5, D6	CXCL11/I-TAC
D7, D8	CXCL13/BLC/BCA-1
D9, D10	CXCL16
D11, D12	Cystatin C

Coordinate	Analyte/Control
D13, D14	DKK-1
D15, D16	DPPIV/CD26
D17, D18	EGF
D19, D20	Endoglin/CD105
D21, D22	Endostatin
D23, D24	Fetuin A/AHSG
E1, E2	FGF acidic
E3, E4	FGF-21
E5, E6	Flt-3 Ligand
E7, E8	Gas 6
E9, E10	G-CSF
E11, E12	GDF-15
E13, E14	GM-CSF
E15, E16	HGF
E17, E18	ICAM-1/CD54
E19, E20	IFN-γ
E21, E22	IGFBP-1
E23, E24	IGFBP-2
F1, F2	IGFBP-3
F3, F4	IGFBP-5
F5, F6	IGFBP-6
F7, F8	IL-1α/IL-1F1
F9, F10	IL-1β/IL-1F2
F11, F12	IL-1ra/IL-1F3
F13, F14	IL-2
F15, F16	IL-3
F17, F18	IL-4
F19, F20	IL-5
F21, F22	IL-6
F23, F24	IL-7
G1, G2	IL-10
G3, G4	IL-11
G5, G6	IL-12 p40
G7, G8	IL-13
G9, G10	IL-15
G11, G12	IL-17A
G13, G14	IL-22
G15, G16	IL-23
G17, G18	IL-27 p28

Coordinate	Analyte/Control
G19, G20	IL-28A/B
G21, G22	IL-33
G23, G24	LDL R
H1, H2	Leptin
H3, H4	LIF
H5, H6	Lipocalin-2/NGAL
H7, H8	LIX
H9, H10	M-CSF
H11, H12	MMP-2
H13, H14	MMP-3
H15, H16	MMP-9
H17, H18	Myeloperoxidase
H19, H20	Osteopontin (OPN)
H21, H22	Osteoprotegerin/TNFRSF11 B
H23, H24	PD-ECGF/Thymidine phosphorylase
I1, I2	PDGF-BB
I3, I4	Pentraxin 2/SAP
I5, I6	Pentraxin 3/TSG-14
I7, I8	Periostin/OSF-2
I9, I10	Pref-1/DLK-1/FA1
I11, I12	Proliferin
I13, I14	Proprotein Convertase 9/PCSK9
I15, I16	RAGE
I17, I18	RBP4
I19, I20	Reg3G
I21, I22	Resistin
J1, J2	Reference Spots
J3, J4	E-Selectin/CD62E
J5, J6	P-Selectin/CD62P
J7, J8	Serpin E1/PAI-1
J9, J10	Serpin F1/PEDF
J11, J12	Thrombopoietin
J13, J14	TIM-1/KIM-1/HAVCR
J15, J16	TNF-α
J17, J18	VCAM-1/CD106
J19, J20	VEGF
J21, J22	WISP-1/CCN4
J23, J24	Negative Control

Figure S6 Multiple cytokines, chemokines, and growth factors were detected in popliteal tissue lysate samples from control, sham injury, injury, and injury plus gel groups of mice (n = 3 in each group) on day 10 after injury.

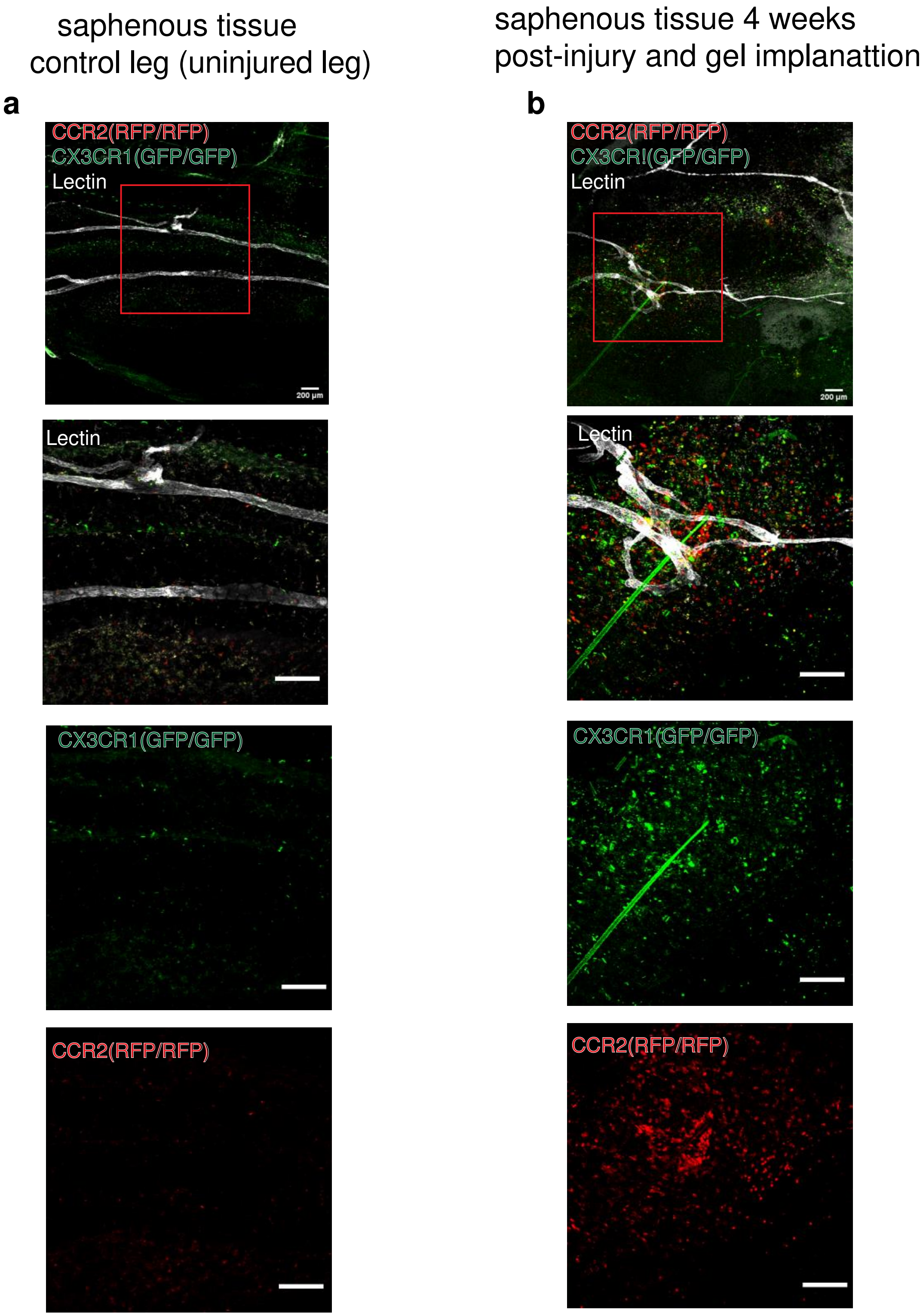

Figure S7 Representative confocal imaging showing infiltration of $CX3CR1^{GFP/GFP}$ and $CCR2^{RFP/RFP}$ cells (functional knockout CX3CR1 and CCR2) 4 weeks after gel implantation in the saphenous tissue. a) Confocal imaging in an uninjured leg indicates the baseline presence of $CX3CR1^{GFP/GFP}$ and $CCR2^{RFP/RFP}$ cells b) Representative imaging shows infiltration of $CX3CR1^{GFP/GFP}$ and $CCR2^{RFP/RFP}$ cells 4 weeks after injury and gel implantation, indicating a deficiency in CX3CR1 or CCR2 does not impede the influx of cells expressing these markers to the injury site. For the sake of imaging, only one lymphatic vessel was injured.

Table S1: The list of antibodies and reagents used in this study. The information from the antibody registry (antibodyregistry.org) database was presented to give a comprehensive list of antibody information and suppliers.

**Antibodies**	**Concentrations**	**Vendor**	**Cat number and registry number**
**CD11c**	**1/200**	**Biolegend**	Cat# 117324, RRID:AB_830649
**CD45**	**1/200**	**Biolegend**	Cat# 103128, RRID:AB_493715
**F4/80**	**1/200**	**Biolegend**	Cat# 123128, RRID:AB_893484
**CCR2**	**1/200**	**R&D**	Cat# FAB5538N, RRID:AB_2725739
**LY6G**	**1/200**	**Biolegend**	Cat# 127608, RRID:AB_1186099
**LY6C**	**1/200**	**Biolegend**	Cat# 128018, RRID:AB_1732082
**Podoplanin**	**1/50**	**DSHB**	DSHB Cat# 8.1.1, RRID:AB_531893
**αSMA**	**1/250**	**Sigma-Aldrich**	Cat# C6198, RRID:AB_476856
**F4/80**	**1/200**	**Bio-Rad**	Cat# MCA497A647, RRID:AB_323931
**Lectin (LEL)**	**1/100**	**Vector Lab**	Cat#DL-1178-1
**CD16/32**	**1/500**	**BioLegend**	Cat# 101301, RRID:AB_312800
**Alexa Fluor 647 Rabbit Anti-Syrian Hamster**	**1/200**	**Jackson ImmunoResearch Labs**	Cat# 307-605-003, RRID:AB_2339601)

Supplementary Videos

Video 1. Time-lapse imaging showing lymphatic sprouting and gel degradation in vitro using lymphatic vessels obtained from an αSMA-DsRed mouse.

Video 2. The procedure for popliteal lymphatic injury.

Video 3. The procedure for fibrin gel implantation after popliteal lymphatic injury.

Video 4. Representative functional NIR imaging prior to surgery. The video framerate has been increased by a factor of ten.

Video 5. Representative functional NIR imaging four weeks post-gel implantation. The video framerate has been increased by a factor of ten.

Video 6. Representative functional NIR imaging four weeks after injury without gel implantation. The video framerate has been increased by a factor of ten.

Supplementary Files

This is a list of supplementary files associated with this preprint. Click to download.

- VideoS1.mp4
- VideoS2.mp4
- VideoS3.mp4
- VideoS4presurgerywithoutgel.mp4
- VideoS5week4withgel.mp4
- VideoS6week4withoutgel.mp4
